# Comparative evaluation of Giomer varnish versus fluoride varnish as desensitizing agents among a group of children with molar incisor hypomineralization: a randomized clinical trial

**DOI:** 10.1186/s12903-026-09760-y

**Published:** 2026-09-15

**Authors:** Ethar Khaled Hassan Swidan, Hala Mohy Eldin Abbas, Shereen Hassan El Shamy

**Affiliations:** Pediatric Dentistry and Dental Public Health, Faculty of Dentistry, Cairo University, New Cairo, Rehab City, Cairo, 11841 Egypt

**Keywords:** Hypersensitivity, Molar Incisor Hypomineralization, Varnish, Desensitizing agents, Oral health-related Quality of life, Pediatric dentistry

## Abstract

**Background:**

Dentin hypersensitivity in children with Molar incisor hypo mineralization (MIH) causes transient sharp pain, adversely affecting oral health- related quality of life (OHRQoL). This research aimed to illustrate the effect of Giomer varnish versus Flouride varnish on hypersensitivity in children with MIH over 4 weeks.

**Methodology:**

Eighty-four children from 8 to 10 years old with a molar incisor hypomineralized hypersensitive permanent first molar were enrolled in the randomized controlled trial study, randomly allocated into two groups, Group I was the experimental group; subjects received Giomer varnish as a desensitizing agent. Group II was the control group, subjects received Flouride varnish as a desensitizing agent. The Schiff scale was used to assess hypersensitivity to evaporative stimulus, while the WONG-BAKER face pain rating scale was used to assess hypersensitivity to thermal and evaporative stimulus. OHRQoL was evaluated before and after treating hypersensitivity using the Arabic version of the Child Perception Questionnaire from 8 to 10 years, with follow up period 4 weeks.

**Results:**

The statistical analysis revealed significant changes between the two groups for the evaluation of pain to evaporative stimulus using the face pain rating scale. After the first week, a statistically significant improvement in hypersensitivity was shown in the experimental group. Another significant difference arose between groups in the first and second weeks in group 1 after evaluating pain to evaporative stimulus using the SCHIFF scale. Evaluating pain response to thermal stimulus showed a significant difference in favor of the experimental group immediately and a week after treatment. Regarding OHRQoL, a significant difference between the two groups was also noticed, with greater improvement in the experimental group.

**Conclusions:**

The Giomer varnish showed significantly greater immediate and sustained reduction in dentin hypersensitivity to both evaporative and thermal stimuli compared to the Fluoride varnish, improving Oral Health Related Quality of Life (OHRQoL), Therefore, the newly introduced Giomer varnish could be a promising desensitizing agent for children suffering from increased hypersensitivity due to MIH.

**Trial registration:**

This clinical trial was registered at [ClinicalTrials.gov] under registration number [NCT05542667] on [13/9/2022].

## Background

Enamel formation is a very sensitive biological process, and any disturbance to the ameloblasts, the cells that produce enamel, can trigger an enamel defect. This may result in fragile and underdeveloped enamel or a normal volume of enamel with inadequate mineralization, depending on the stage of the ameloblasts' life cycle disrupted. When discussing insufficient mineralization, it can incorporate enamel defects like Molar Incisor Hypomineralization (MIH), Hypomineralized Second Primary Molar (HPSM), and Deciduous Molar Hypomineralization (DMH) [1].

In comparison with sound teeth, those with enamel defects demonstrate poorly defined prism sheaths and a diminished content of hydroxyapatite crystals. Consequently, the hypomineralized enamel exhibits lower mechanical characteristics, such as elasticity and hardness, than normal enamel. Additionally, the enamel affected by these conditions demonstrates higher levels of proteins, including serum albumin, alpha-1 antitrypsin, antithrombin III, type I collagen, and ameloblastin, which hinder the growth of hydroxyapatite crystals, leading to reduced enamel quality minerals [1].

The prevalence of Molar Incisor Hypomineralization differs, but approximately 27.4% of all individuals with MIH will require intervention to restore teeth for daily functional use. Therefore, with approximately 5 million new cases of MIH identified/diagnosed annually, there are approximately 240 million existing MIH cases without treatment; many are untreated in countries with lower socioeconomic status, with a global incidence of MIH equal to approximately 14.2% [2].

Hypersensitivity, decay following eruption, and other dental problems that are associated with MIH present a variety of difficult clinical challenges. Furthermore, children with MIH experience poor aesthetics and impact their health and well-being through reduced quality of life (QoL). There are several challenges involved in treating MIH-affected teeth for clinicians, such as how to manage pain, what restoration material to use, and where to finish the borders of the restoration [3].

Casein-derived products, as well as fluoride varnishes (FVs), are now regarded as an effective protective and reparative measure when treating MIH. Fluoride Varnish promotes enamel remineralization through the encouragement of calcium and phosphate ions from within the tooth, which increases enamel resistance to caries and sensitivity, while also creating a temporary barrier on the surface of each compromised site to slow enamel degradation and enhance the uptake of fluoride by the tooth's enamel over time [4].

Casein phosphopeptide-amorphous calcium phosphate (CPP-ACP), which is a casein-derived material, has also been found useful for managing MIH. With respect to the management of MIH, CPP-ACP maintains a sufficient level of calcium and phosphate ions at the surface of the tooth such that the remineralization process is optimized and ongoing enamel breakdown is prevented. Together, casein-derived products and FVs will provide a complete approach to halting the progression of MIH and assisting with the restoration of the structural integrity of affected teeth [4].

One of the newest treatment options, S-PRG/Giomer technology, is an approach that features multi-ionic trilaminar glass particles, called S-PRG (surface pre-reacted glass-ionomer “PRG”). As a xerogel, its outer porous layer helps release multiple ions, including fluoride, strontium, silicate, aluminum, boron, and sodium. In the commercial market, it's introduced as PRG Barrier Coat, a light-cured varnish used as an adhesive coating layer. When smeared to the enamel surface, it's principally used as a dentin hypersensitivity material and to remineralize areas prone to dental caries [5].

Molar Incisor Hypomineralization is considered a challenge for patients, parents, and even pediatric dentists. Many treatment options have been proposed, including Giomer varnish, which is a newly introduced product to the market and has shown many superior results. Due to its superior properties, it requires further research, especially regarding its use in pediatric dentistry, as there are currently only a few studies conducted on pediatric patients. The manufacturer introduced the PRG BARRIER COAT as a desensitizing agent. Therefore, this study focused on comparing the effects of the newly introduced Giomer varnish versus the conventionally used fluoride varnish in treating hypersensitivity in MIH patients.

## Materials and methods

### Study design

**Fig. 1:**
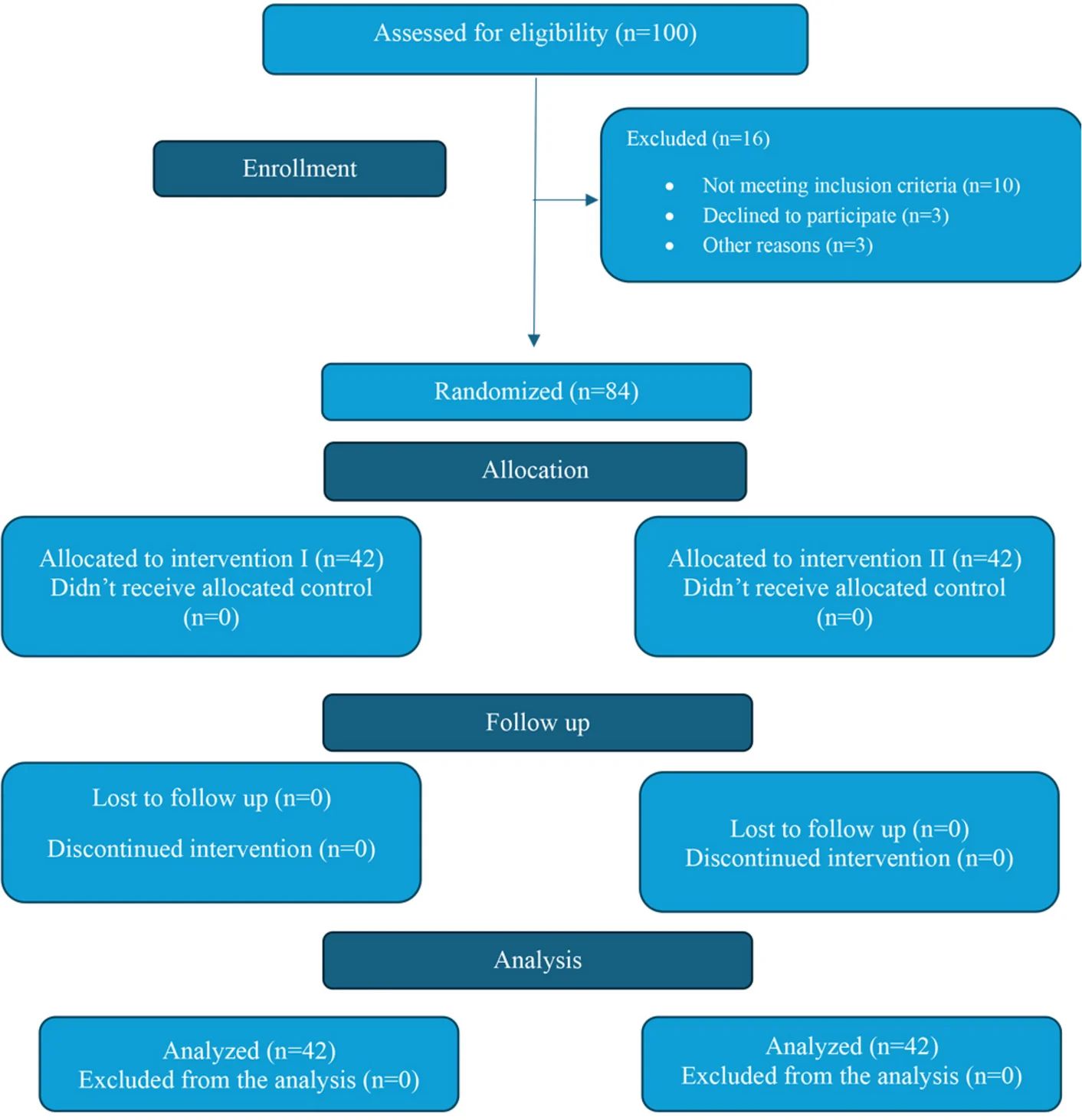
CONSORT flow diagram

This was a randomized, double-blind, two-parallel-arm clinical trial at Cairo University’s Faculty of Dentistry. The research plan adhered to the guidelines instructed in the Consolidated Standards of Reporting Trials (CONSORT) statement (Fig. 1).

The following published trial adheres to the CONSORT guidelines for reporting.

### Ethical approval and trial registration

The Ethical Committee, Faculty of Dentistry, Cairo University, revised and approved the work protocol for its scientific content and compliance with research ethics and human subject regulations (Code: 17,123). This clinical trial was registered at [ClinicalTrials.gov] under registration number [NCT05542667] on [13/9/2022].

### Sample size calculation

A power analysis was organized to have adequate power to employ a statistical test of the null hypothesis that there is no difference between tested groups. By assuming α level of (0.05) i.e. power = 80% and an effect size (d) of (0.682) estimated based on the findings of past research [6] and on expert’s opinion; the predicted sample size (n) was found to be 35 cases/group (a total 70 cases). Sample size was raised by (20%) to account for possible dropouts to be 42 cases/group (a total 84 cases). Sample size calculation was conducted via PS Power and Sample Size Calculations version 3.1.2.

### Participant eligibility criteria

All participants were examined and enrolled in this study according to predetermined inclusion and exclusion criteria was demonstrated in Table 1.

**Table 1:** Inclusion and exclusion criteria

**Inclusion criteria for participants**	**Exclusion criteria for participants**
1. Patients aged between 8 and 10 years old with MIH affected teeth according to European Academy of Pediatric Dentistry diagnostic criteria 2. Systemically healthy 3. Cooperative patients who will comply with follow-up visits	1. Undergoing desensitizing treatments in the last 3 months 2. Occlusal problems such as bruxism 3. Cognition problems 4. Who used any type of analgesics before treatment 5. Undergoing orthodontic treatment

**Inclusion criteria for teeth**	**Exclusion criteria for teeth**
1. At least one first molar or one incisor erupted in the oral cavity exhibiting molar incisor hypomineralization 2. Teeth representing molar incisor hypomineralization are sensitive	1. Enamel defects, such as enamel hypoplasia, amelogenesis imperfecta, and dental fluorosis 2. Carious teeth

Children aged 8–10 years were selected because this age range corresponds to the recommended period for reliable MIH diagnosis according to the European Academy of Pediatric Dentistry (EAPD), as the first permanent molars are fully erupted and can be clinically assessed. Furthermore, children in this age group are generally capable of providing consistent responses to validated pain assessment tools. Previous MIH studies have similarly focused on children around 8–9 years of age. Although this age range may limit generalizability to all pediatric populations, it was intentionally selected to ensure diagnostic accuracy and reduce age-related variability [7, 8].

### Recruitment and informed consent

Personal, dental, and medical histories of each participant were recorded prior to clinical exams. All participants had comprehensive study information about goals, methods, potential benefits/risks, expected duration of participation, and safety measures. Guardians of the eligible subjects were then needed to sign an Arabic informed consent, authorized by Cairo University’s Faculty of Dentistry Research Ethical Committee (REC), describing the trial’s ethical considerations and confirming voluntary contribution.

### Group allocation

Contributors were randomized into two groups—Group I (Giomer varnish) and Group II (Flouride Varnish).

### Randomization

The subjects were randomly assigned to receive the treatment, either Giomer varnish or Fluoride varnish. using a random order generator (http://www.random.org/) to apply random sequencing of the patients into the intervention’s groups with allocation ratio 1:1 according to block randomization.

### Allocation concealment

Folded papers carrying information about which intervention to be used and to which tooth, were packed in opaque sealed envelopes. Then, these envelopes were delivered to the operator by the patients.

### Blinding

Single—blinded study; the operator could not be blinded due to the nature of the study, but Participants and the statistician were blinded to the agents used in the study to avoid reporting bias.

The CONSORT flow diagram illustrates the numbers of participants assessed for recruitment, eligibility, allocation, randomization, and analysis (Fig. 1).

### Materials

The materials applied in this study were Giomer varnish, PRG Barrier coat by Shofu PRG Barrier Coat (Shofu Inc., Kyoto, Japan) and Fluoride varnish, Biflourid 10 by Voco Bifluorid 10 (VOCO GmbH, Cuxhaven, Germany). A full detailed description of the materials is illustrated in Table 2.

**Table 2:** Materials used in the trial

**Material**	**Specification**	**Composition**	**Manufacturer**	**LOT#**
PRG Barrier Coat	BioSmart Light Cured Protective Shield with bioactive S-PRG filler technology	Base: S-PRG filler based on fluoroboroaluminosilicate glass, distilled water, Methacrylic acid monomer, and others Active: Phosphonic acid monomer, Methacrylic acid monomer, Bis-MPEPP, Carboxylic acid monomer, TEGDMA, Polymerization initiator, and others	SHOFU, Kyoto, Japan	04230
Biflourid 10	Fluoride varnish (Desensitizing agent) containing 5% NaF (22,600 ppm fluoride) + 5% CaF₂	Active: Sodium fluoride 5%, Calcium fluoride 5%, Synthetic resin base (transparent varnish matrix), solvent system (organic solvents), flavoring agents Key feature: dual fluoride release (immediate + long-term)	VOCO GmbH, Cuxhaven, Germany	2345240

1. Bis-MPEPP: 2,2-bis (4-methacryloxy polyethoxyphenyl) propane

2. TEGDMA: Triethylene glycol dimethacrylate

3. NaF: Sodium fluoride

4. CaF₂: Calcium flouride

### Harms related to varnish application and management of these harms

- Direct contact of the varnish to the tongue, oral mucosa, or gingiva causing erythema. Any tissues that come into contact with the varnish should be wiped with cotton containing alcohol and then rinsed with water according to manufacturer instructions.
- Injury or abrasion of the gingiva from the polishing brush used while cleaning the teeth, and risk of swallowing Fluoride varnish, causing gastric upset. Apply any gingival barrier during the application of varnish or teeth polishing to prevent injury to gingival tissues.
- Failure of both varnishes to decrease hypersensitivity; use an alternative technique to ensure reduction in hypersensitivity

### Clinical procedure

#### Patients’ preparation

Personal information, including name, age, phone number, address, medical history, and dental history, was collected by the operator from each patient in special forms. History of chief complaint was obtained.

The teeth were examined tentatively via a diagnostic mirror and probe for the existence of extensive caries, large or old restorations, pulp exposure, and soft tissue. Tooth mobility as well as percussion and palpation were done to confirm that no mobility or pain on palpation were present, as well as no or slight discomfort on percussion. Visual inspection of the face and neck was performed to exclude the presence of any swellings.

**Fig. 2:**
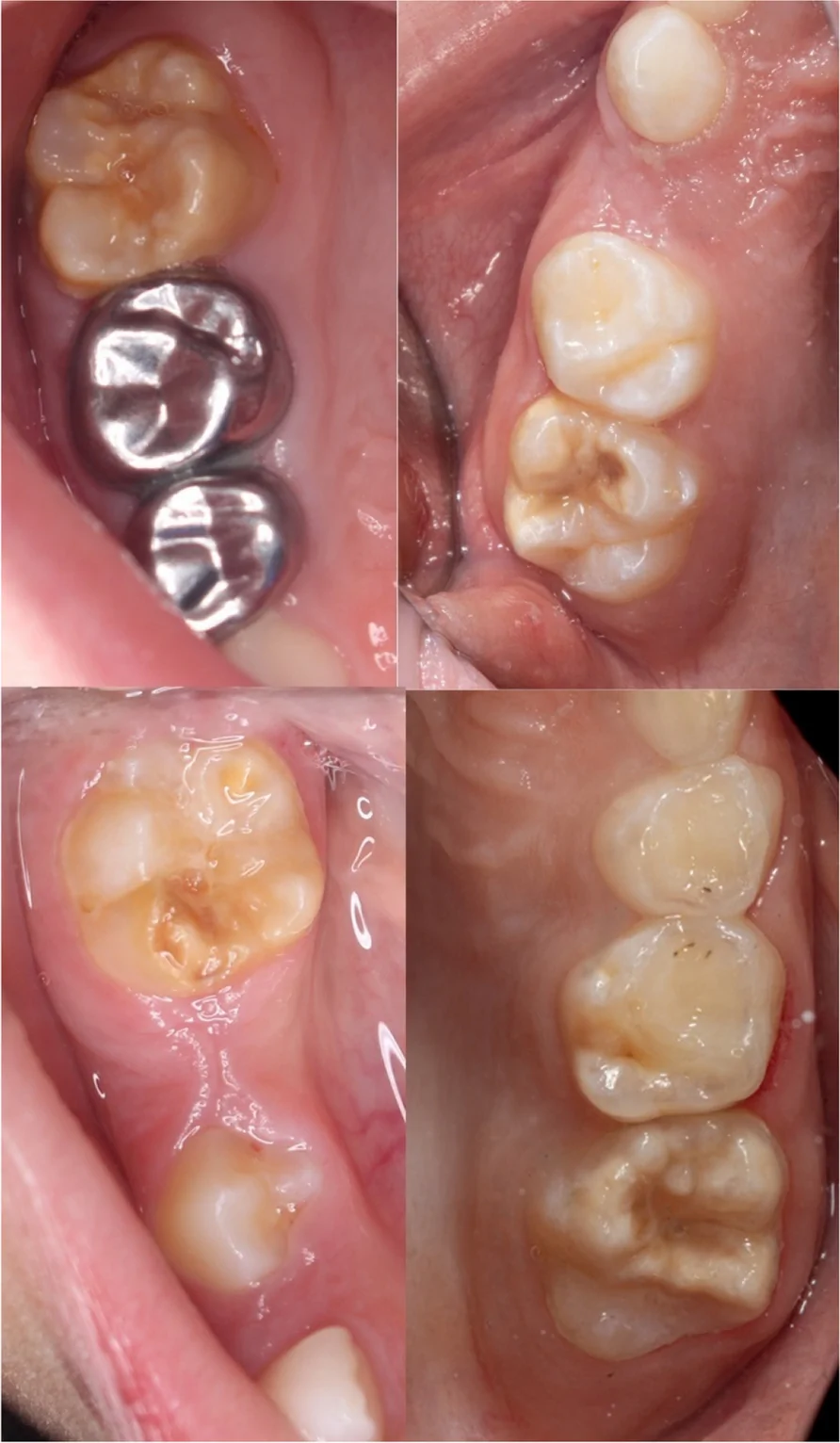
Intra oral clinical photos

Teeth with a final diagnosis of Molar Incisor Hypomineralization were chosen for the study based on the History of chief complaint reporting pain with hot or cold stimulus. All harms and benefits of the treatment were explained to the parents and post -treatment instructions were explained and informed consent was signed. After enrollment of patients, pre-clinical intra-oral photographs were taken prior to treatment (Fig. 2).

### Dentin hypersensitivity assessment

#### Evaporative stimulus

**Fig. 3:**
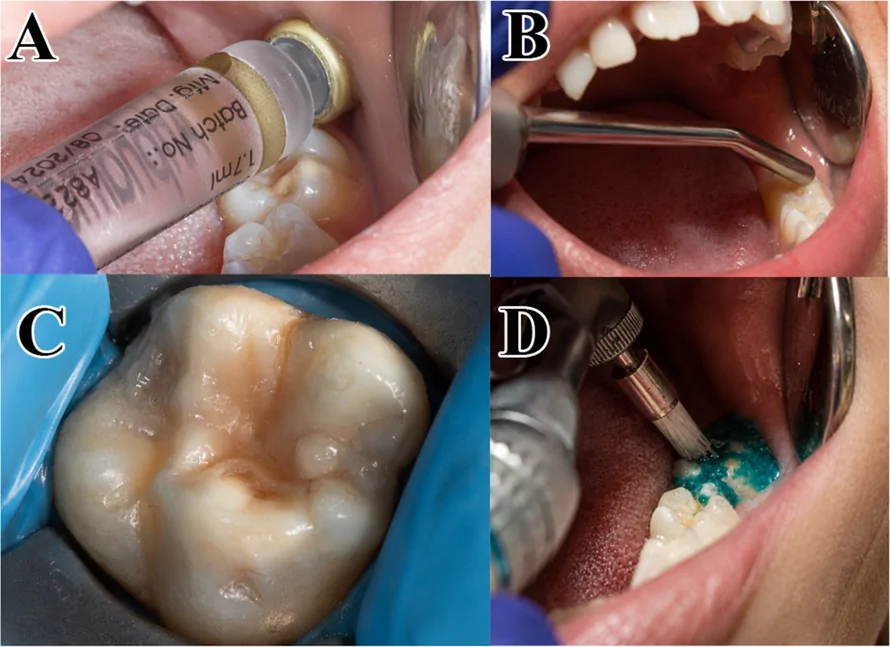
Stimuli application and tooth preparation **A**: using thermal stimulus, **B** using evaporative stimulus, **C** rubber dam application, **D** cleaning tooth

**Fig. 4:**
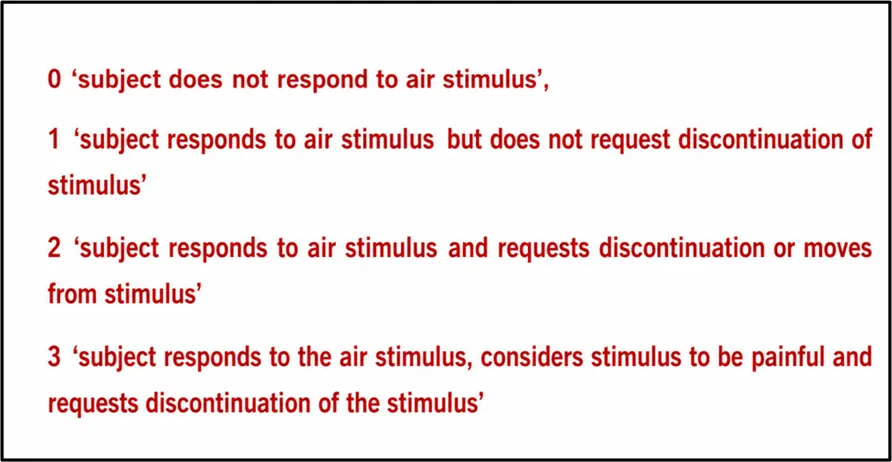
SCHIFF scale

**Fig. 5:**
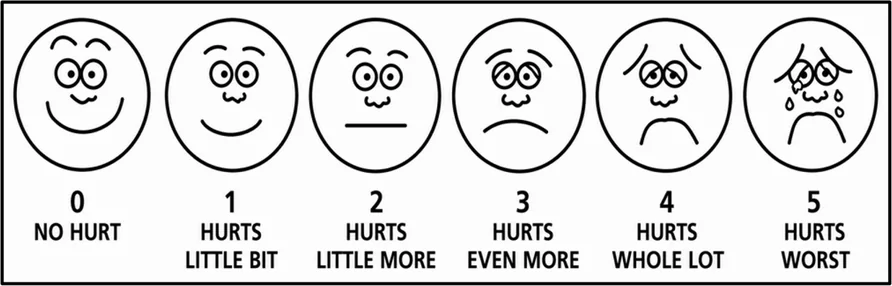
Wong baker face pain rating scale

All included teeth were assessed for pain due to an evaporative stimulus applying a triple syringe (Fig. 3). The air is focused perpendicular to the teeth and persists about 2 mm away for 3 s. Children are instructed to raise their fingers or hands if they experience any distress, and then the pain response is estimated using the Schiff scale demonstrated in (Fig. 4) and Wong-Baker Faces Pain Scale illustrated in (Fig. 5) [6].

### Thermal stimulus

An anesthetic carpule, refrigerated at 4 °C for at least 24 h, was put in contact with the tooth for 1 to 5 s (Fig. 3), depending on the child response. Pain was assessed with Wong baker FACES scale (Fig. 5) [7].

### Oral health-related quality of life assessment

**Fig. 6:**
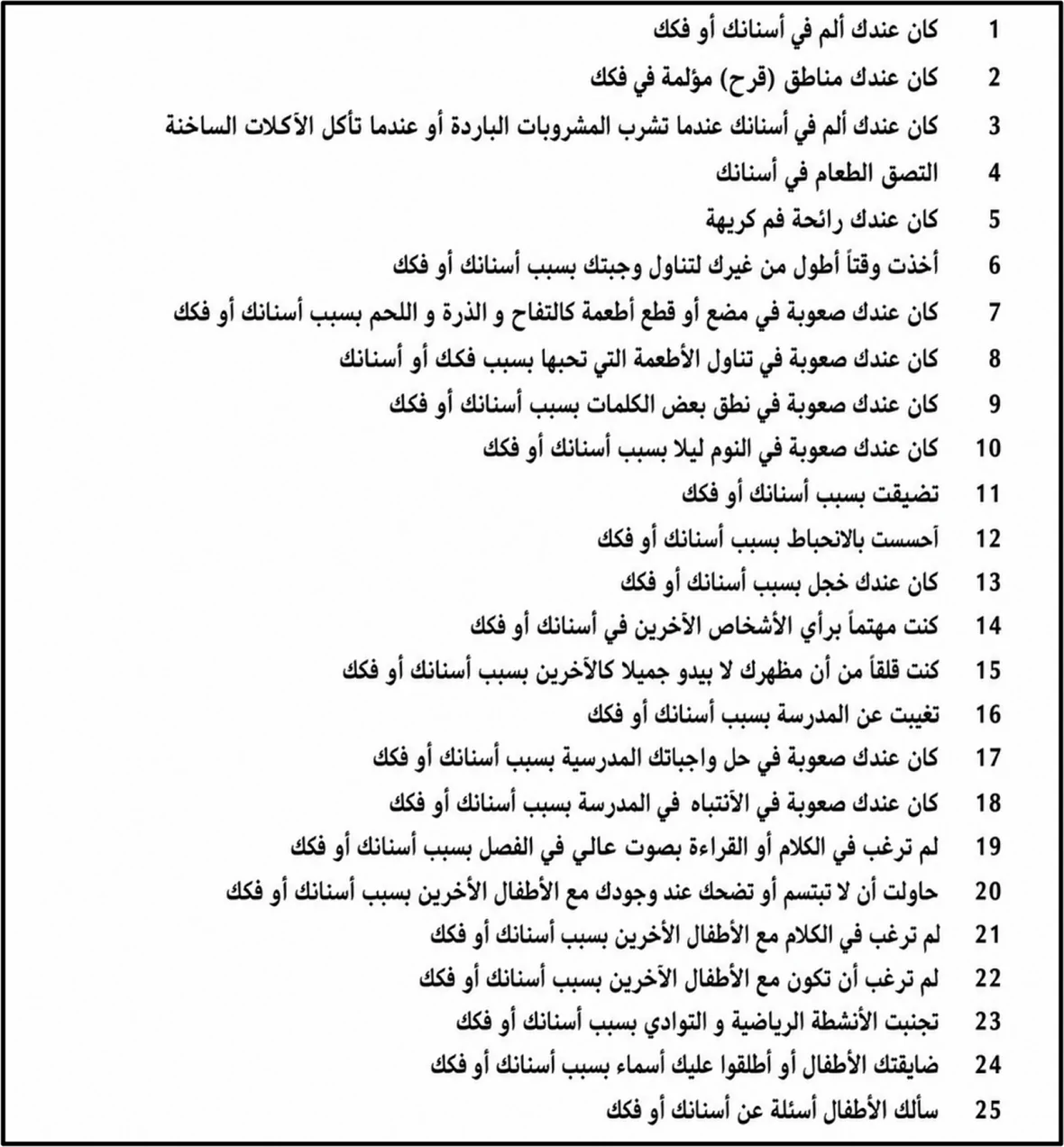
Arabic version of child perception questionnaire

Oral health-related quality of life was evaluated in this study by employing the Arabic version of CPQ from 8 to 10 years (Fig. 6).

### Preparation phase

#### Prohylaxsis

Pre-treatment prophylaxis was performed via a fluoride-free paste (Quartz, Dharma Research Inc., USA) applied with a nylon polishing brush (Microdont, São Paulo, SP, Brazil) attached to a low-speed handpiece (Contra T4 HP, Sirona, Germany). The tooth surfaces were then thoroughly cleaned with an air–water spray and gently dried with cotton pellets to get rid of debris and moisture, confirming optimal surface preparation.

### Isolation

Isolation was performed via rubber dam application on selected molar (KSK molar clamp no 202, Dentech, Japan) (Fig. 3).

### Treatment phase

#### Material application

**Fig. 7:**
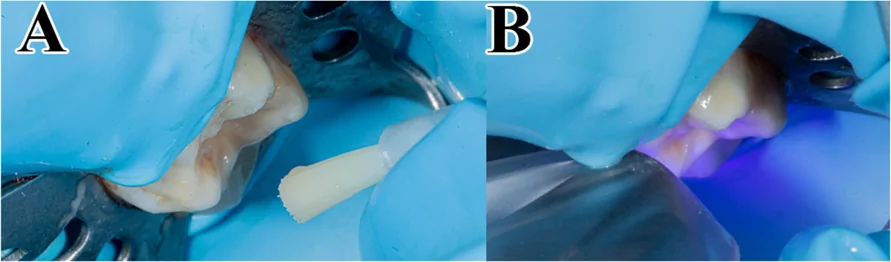
Giomer varnish application on upper permanent first molar, **A** application of Giomer varnish with its applicator, **B** light curing of the varnish

For Group I, Giomer varnish was applied once during the initial treatment visit. One drop of PRG Coat Barrier ACTIVE was added to the container supplied in the kit containing PRG Coat Barrier BASE. The components were mixed using the disposable mixing tip provided by the manufacturer. A thin layer was then applied and set undisturbed for 3 s as stated by the manufacturer’s guidelines. Finally, the varnish was light-cured using a Woodpecker curing light (Woodpecker Medical Instrument Co., Guilin, China) (Fig. 7) [7].

**Fig. 8:**
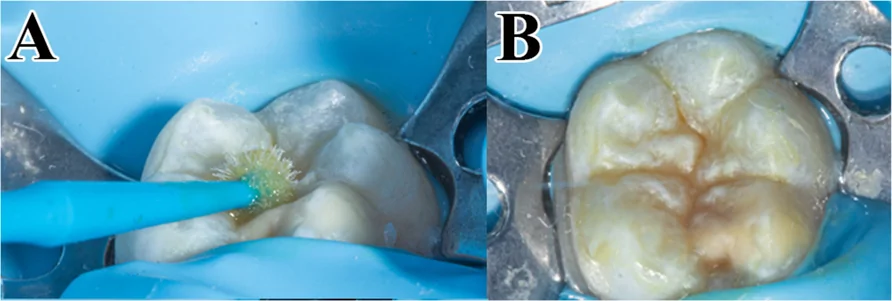
Biflourid 10 varnish application on a lower first permanent molar, **A** fluoride varnish application with micro brush, **B** Yellowish discoloration of the tooth after application of fluoride varnish

For the Group II Fluoride varnish group, the seal of the single-use Bifluorid 10 vial was pierced via a Micro-Tim brush. A thin layer of the varnish was then employed with a microbrush over four sessions, at a frequency of one session per week. The varnish was left undisturbed for 10–20 s and subsequently air-dried, as stated by the manufacturer’s guidelines (Fig. 8). Reapplication of the varnish at each weekly follow up visits according to manufacturer instructions [7].

The application protocols were selected according to the manufacturers’ instructions for use rather than being determined by the investigators. Giomer varnish (PRG Barrier Coat,Shofu) is intended as a single-application, light-cured protective coating providing prolonged protection up to 6 months according to manufacturer’s instructions and hypersensitivity relief, with reapplication performed only when clinically indicated. In contrast, the manufacturer’s instructions for Fluoride varnish (Bifluorid 10,VOCO) recommend repeating the treatment at 7-day intervals for hypersensitivity management. Therefore, the difference in application frequency reflects the intended use of each material and represents their real-world clinical utilization [9].

Patients are to be instructed not to brush or eat hard food for at least 4 h after varnish application. And restricted from eating any staining food for 3 to 4 days after application of the varnish.

Any other dental problems rather than teeth involved in the study were treated during the follow-up period, including any extractions, restorations, pulp treatment, and full coverage. These treatments were done in both groups for all participants.

### Outcomes measures

#### Primary outcome

Pain to evaporative stimulus was measured used SCHIFF scale and Wong baker face pain rating tool at baseline, 1 week, 2 weeks, 3 weeks and 4 weeks.

#### Secondary outcomes

Pain to thermal stimulus was rated using the Wong Baker FACES pain rating scale at baseline, 1 week, 2 weeks, 3 weeks, and 4 weeks. Oral health-related quality of life was evaluated via CPQ8-10 at baseline and 4 weeks.

### Follow-up procedures

Patients were recalled each week to assess dentin hypersensitivity under standard conditions, and intra-oral clinical photographs were taken at each follow-up visit.

The primary outcome of the present study was the reduction of hypersensitivity, which is considered an early clinical outcome. Previous studies evaluating hypersensitivity in MIH-affected molars have included assessments at 1 and 4 weeks, supporting the clinical relevance of short-term follow-up periods. Therefore, a 4-week follow-up was considered appropriate to evaluate the initial efficacy of the investigated varnishes [10].

### Statistical analysis

Statistical analysis was conducted with SPSS 27®, Microsoft Excel 2016, and Graph Pad Prism®. All results were verified for normality using Shapiro Wilk Normality test Kolmogorov tests. Which illustrated that all data except age originated from non-parametric origin. Comparison between both groups was done via Mann Whitney test, while comparison between different intervals was carried out with Friedman test and subsequently Dunn`s test multiple comparisons. In age, comparison between groups was done with independent tests. In categorical results, all comparisons were accomplished via Chi square test and fishers exact test. The significant level was determined to be at *P* ≤ *0.05*.

## Results

### Baseline data

Demographic data of the groups were presented in (Tables 3 and 4). Comparison between groups revealed that both groups were demographically comparable, with no statistically significant changes in sex distribution, age, or tooth numbers. (all *p* > 0.05).

**Table 3:** Demographic data of both groups

		**Group I Experimental**	**Group II Control**	***P* value**
Age M ± SD		8.93 ± 0.89	9.12 ± 0.86	0.32
Tooth number N (%)	**16.00**	11 (26.2%)	15 (35.7%)	0.61
	**26.00**	10 (23.8%)	12 (28.6%)	
	**36.00**	11 (26.2%)	8 (19.0%)	
	**46.00**	10 (23.8%)	7 (16.7%)	
Sex N(%)	**Male**	19 (45.2%)	16 (38.1%)	0.51
	**Female**	23 (54.8%)	26 (61.9%)

**Table 4:** Dental examination of both groups

		**Minimum**	**Median**	**Maximum**	**Mean**	**Standard Deviation**	***P* value**
DMF	**Group I Experimental**	0.00	1.00	4.00	1.02	1.12	0.26
	**Group II Control**	0.00	0.00	3.00	0.83	1.15	
def	**Group I Experimental**	0.00	2.50	7.00	2.71	1.93	0.23
	**Group II Control**	0.00	2.00	10.00	2.31	2.53	
dmf	**Group I Experimental**	0.00	0.00	0.00	0.00	0.00	0.32
	**Group II Control**	0.00	0.00	1.00	0.02	0.15	

### Primary outcome

#### Absence of sensitivity to evaporative stimulation (SCHIFF SCALE)

Comparison between groups regarding sensitivity to evaporative stimulation showed that both groups started with identical baseline values, with no significant difference (*P* = 0.939) with the same median score and identical mean values (1.90 ± 0.79 in Group I and 1.90 ± 0.91 in Group II). Immediately after treatment, sensitivity decreased in both groups, but the difference remained non-significant (*P* = 0.065). Despite lower mean scores in Group I (1.02 ± 0.84) than Group II (1.45 ± 1.02), the change did not yet reach statistical significance. A significant intergroup difference appeared at 1 week (*P* = 0.002)when Group I showed a substantially lower pain response (0.45 ± 0.80) compared with Group II (1.14 ± 1.12). This significant difference persisted at two weeks (*P* = 0.006), with Group I again demonstrating much lower sensitivity (0.43 ± 0.70 vs. 1.02 ± 1.07 in Group II), with the experimental group showing a markedly greater reduction in sensitivity. By 3 and 4 weeks, Group I continued to demonstrate lower scores than Group II; conversely, these differences were no longer statistically meaningful (*P* = *0.117* and *P* = *0.088*)). Despite this, the results favored Group I, which maintained mean scores below 0.5 across all later follow-up points, compared with higher and more variable scores in the control group. Overall, the experimental group showed a faster and more pronounced early reduction in thermal sensitivity, while both groups exhibited a gradual decline over time.

At one week, a statistically significant intergroup difference was observed (χ2 = 10.33, *p* = 0.016), with a moderate effect size (Cramér’s V = 0.35), indicating a clinically meaningful association between treatment allocation and evaporative sensitivity scores.

Within both groups, sensitivity to evaporative stimulus showed a highly significant reduction over time (*P* < 0.0001). In Group I, SCHIFF scores dropped rapidly after treatment and remained consistently low throughout follow-up, with no further significant changes between post-treatment time points. Group II also showed a significant gradual decrease over time, but the reduction was less marked, and sensitivity remained higher than in Group I at most evaluations. Overall, both groups improved significantly, with a faster and more stable response observed in the experimental group.

All of these findings are illustrated in Table 5.

**Table 5:** Comparison between groups regarding tooth Evaporite stimulus using SCHIFF scale

**Evaporite stimulus using SCHIFF scale**	**Group I (Experimental)**		**Group II (Control)**		***P* value**
	**Min/Max (Median)**	**M ± SD**	**Min/Max (Median)**	**M ± SD**	
Before TTT	1/3 (2)	1.90 ± 0.79^**a**^	1/3 (2)	1.90 ± 0.91^**a**^	0.939
After TTT	0/3 (1)	1.02 ± 0.84^**b**^	0/3 (1)	1.45 ± 1.02^**ab**^	0.065
After 1 week	0/3 (0)	0.45 ± 0.80^**b**^	0/3 (1)	1.14 ± 1.12^**bc**^	0.002*
After 2 weeks	0/3 (0)	0.43 ± 0.70^**b**^	0/3 (1)	1.02 ± 1.07^**bc**^	0.006*
After 3 weeks	0/2 (0)	0.48 ± 0.67^**b**^	0/3 (0.5)	0.90 ± 1.12^**bc**^	0.117
After 4 weeks	0/2 (0)	0.43 ± 0.67^**b**^	0/3 (0)	0.86 ± 1.09^**c**^	0.088
*P* value	< 0.0001*		< 0.0001*		

^*^Significant difference as *P* ≤ 0.05

Means with different superscript letters were significantly different as *P* < 0.05

#### Absence of pain to evaporative stimulus using face pain rating scale

Comparison between groups regarding evaporative stimulation sensitivity (Face Rating Scale) showed no significant difference at baseline (*P* = *0.343*), indicating comparable initial pain levels. Immediately after treatment, a significant intergroup difference emerged (*P* = *0.032*), with Group I showing lower pain scores than Group II. This significant advantage for Group I persisted at 1 and 2 weeks (*P* = *0.017* and *P* = *0.042*), while at 3 and 4 weeks no significant differences were observed, although Group I consistently maintained lower scores. At baseline, the mean pain scores for evaporative stimulation were 2.50 ± 1.13 and 2.76 ± 1.32 in Group I and Group II, respectively. By the 4-week follow-up, both groups demonstrated a marked reduction in hypersensitivity, with mean scores decreasing to 0.83 ± 1.10 in Group I and 1.48 ± 1.71 in Group II, indicating sustained improvement over the study period..

The intervention demonstrated its greatest clinical effect immediately after application (Cramér’s V = 0.43), with a sustained moderate effect observed at one week (Cramér’s V = 0.39). Thereafter, the magnitude of the treatment effect gradually decreased over the follow-up period.

In both groups, there was a significant decrease in pain to evaporative stimulation (Face Rating Scale) with time (*P* < *0.05*). For Group I, there was a rapid decrease following treatment which was maintained consistently without any significant difference between the post-treatment follow-up period). The mean pain score decreased significantly from 2.50 ± 1.13 before TTT to 1.10 ± 1.25 immediately after TTT, and this reduction was maintained at 1 week (0.93 ± 1.05), 2 weeks (0.90 ± 1.08), 3 weeks (0.98 ± 1.09), and 4 weeks (0.83 ± 1.10). There was a progressive yet significant reduction in Group II; the mean pain score decreased from 2.76 ± 1.32 before TTT to 1.88 ± 1.71 after TTT, with further reductions observed at 1 week (1.76 ± 1.64), 2 weeks (1.62 ± 1.59), 3 weeks (1.52 ± 1.70), and 4 weeks (1.48 ± 1.71). However, with relatively higher pain scores than Group I at each follow-up point. Statistical data are illustrated in (Table 6).

**Table 6:** Comparison between groups regarding sensitivity to evaporative stimulation (Face rating scale)

**pain response to the evaporative stimulation (Face rating scale)**	**Group I (Experimental)**		**Group II (Control)**		***P* value**
	***Min/Max (Median)***	***M* ± *SD***	***Min/Max (Median)***	***M* ± *SD***	
Before TTT	1/5 (2)	2.50 ± 1.13^**a**^	1/5 (3)	2.76 ± 1.32^**a**^	0.343
After TTT	0/4 (1)	1.10 ± 1.25^**b**^	0/5 (1)	1.88 ± 1.71^**b**^	0.032*
After 1 week	0/4 (1)	0.93 ± 1.05^**b**^	0/5 (1.5)	1.76 ± 1.64^**b**^	0.017*
After 2 weeks	0/4 (1)	0.90 ± 1.08^**b**^	0/5 (1)	1.62 ± 1.59^**b**^	0.042*
After 3 weeks	0/4 (1)	0.98 ± 1.09^**b**^	0/5 (1)	1.52 ± 1.70^**b**^	0.277
After 4 weeks	0/4 (0.5)	0.83 ± 1.10^**b**^	0/5 (1)	1.48 ± 1.71^**b**^	0.142

^*^Significant difference as *P* ≤ 0.05

Means with different superscript letters were significantly different as *P* < 0.05

### Secondary outcomes

#### Pain response to thermal stimulation (using only Face rating scale)

Using the Mann–Whitney test to compare intergroup differences at the start of the study, we found that there were no statistical differences in tooth sensitivity between groups (*P* = *0.450*). Similarly, tooth sensitivity did not differ between groups immediately after treatment (*P* = *0.128*). However, there was a statistically significant difference at one week (*P* = *0.028*) and at four weeks (*P* = *0.011*) with consistently lower ratings reported for the experimental group compared to the control group. Differences in tooth sensitivity were not statistically significant between groups at two and three weeks, although the experimental group had lower mean tooth sensitivity ratings than the control group.

For thermal stimulation, the baseline mean pain scores were 2.43 ± 1.47 for Group I and 2.21 ± 1.44 for Group II. At 4 weeks, mean scores decreased to 0.57 ± 1.11 and 1.31 ± 1.55 in Group I and Group II, respectively, demonstrating a persistent reduction in thermal hypersensitivity in both treatment groups throughout the follow-up period.

Although no statistically significant intergroup differences were observed for thermal sensitivity at any time point, effect size analysis demonstrated small-to-moderate treatment effects across the follow-up period (Cramér’s V range: 0.20–0.31), suggesting that the intervention may have had a clinically relevant impact that warrants further investigation in studies with larger sample sizes.

The intra-group analysis conducted using the Friedman test showed a statistically significant decrease in tooth sensitivity across all groups at all time points (*p* < *0.0001*). The experimental group showed an immediate decrease in tooth sensitivity after treatment that continued to decrease over time and remained low at all follow-up visits, from a baseline mean of 2.43 ± 1.47 to 1.26 ± 1.33 immediately after treatment, and continued to fall to less than 1.0 from the first week onward. Median values also dropped from 2 at baseline to 0 starting from the first week. The control group also showed a significant gradual reduction over time, with mean sensitivity scores declining from 2.21 ± 1.44 at baseline to 1.76 ± 1.54 after treatment, and continuing to decrease through the 4-week period, but the improvement was less pronounced, and sensitivity levels remained higher compared to the experimental group at most time points. All these findings are demonstrated in (Table 7).

**Table 7:** Comparison between groups regarding tooth sensitivity to thermal stimuli using face rating scale

**thermal stimulus (face pain rating scale)**	**Group I (Experimental)**		**Group II (Control)**		***P* value**
	**Min/Max (Median)**	**M ± SD**	**Min/Max (Median)**	**M ± SD**	
Before TTT	1/5 (2)	2.43 ± 1.47^**a**^	1/5 (2)	2.21 ± 1.44^**a**^	0.450
After TTT	0/4 (1)	1.26 ± 1.33^**b**^	0/5 (1.5)	1.76 ± 1.54^**ab**^	0.128
After 1 week	0/4 (0)	0.62 ± 1.15^**b**^	0/5 (1)	1.33 ± 1.68^**b**^	0.028*
After 2 weeks	0/5 (0)	0.90 ± 1.25^**b**^	0/5 (0.5)	1.33 ± 1.71^**b**^	0.406
After 3 weeks	0/5 (0)	0.67 ± 1.12^**b**^	0/5 (0)	1.05 ± 1.43^**b**^	0.245
After 4 weeks	0/5 (0)	0.57 ± 1.11^**b**^	0/5 (1)	1.31 ± 1.55^**b**^	0.011
*P* value	< 0.0001*		< 0.0001*		

^*^Significant difference as *P* ≤ 0.05

Means with different superscript letters were significantly different as *P* < 0.05

### Oral health-related quality of life:

Comparison between groups regarding changes in OHRQoL showed that, at the 1 st visit, there were no remarkable changes between either group in oral symptoms, functional limitation, or emotional well-being (*P* > *0.05*). However, Group I showed significantly better outcomes in social well-being (*P* = *0.001*) and a significantly lower total CPQ score (*P* = *0.02*) compared with Group II, indicating an overall better QoL early in the study. At the last visit, no significant differences were observed between groups in functional limitation, oral symptoms, or emotional well-being (*P* > *0.05*), while Group I continued to show significantly better social well-being (*P* = *0.003*) and a reduced total CPQ score (*P* = *0.003*), reflecting a sustained overall advantage in OHRQoL in the experimental group.

Within both groups, OHRQoL improved significantly from the 1 st to the last visit (P < 0.001). In Group I, all domains and the total CPQ score showed a marked reduction, indicating a strong overall improvement. Similarly, Group II demonstrated significant improvements across all domains, although the degree of improvement was slightly greater in the experimental group.

Intergroup and intra-group comparisons are shown in (Tables 8 and 9).

**Table 8:** Comparison between groups regarding patients oral health related quality of life

		**Group I (Experimental)**		**Group II (Control)**		***P* value**
		**Min/Max (Median)**	**Mean ± SD**	**Min/Max (Median)**	**Mean ± SD**	
1 st visit	**oral symptoms**	0.40/3.00 (1.80)	1.78 ± 0.75	0.00/3.00 (2.00)	1.70 ± 0.89	0.996
	**functional limitation**	0.00/3.00 (1.00)	1.07 ± 0.73	0.00/3.40 (1.10)	1.35 ± 0.88	0.143
	**emotional well being**	0.00/4.00 (0.80)	1.05 ± 0.93	0.00/3.80 (1.20)	1.35 ± 0.91	0.116
	**social well being**	0.00/2.50 (0.60)	0.77 ± 0.69	0.10/2.60 (1.35)	1.28 ± 0.71	0.001*
	**CPQ sum**	0.80/8.70 (4.65)	4.67 ± 2.04	2.10/8.80 (5.50)	5.69 ± 1.70	0.02*
Last visit	**oral symptoms**	0.00/2.40 (1.00)	1.03 ± 0.53	0.00/2.60 (1.20)	1.11 ± 0.73	0.434
	**functional limitation**	0.00/2.00 (0.60)	0.66 ± 0.53	0.00/2.60 (0.80)	0.92 ± 0.72	0.130
	**emotional well being**	0.00/2.20 (0.60)	0.69 ± 0.62	0.00/2.40 (0.90)	0.84 ± 0.66	0.250
	**social well being**	0.00/1.90 (0.40)	0.52 ± 0.52	0.00/2.00 (0.90)	0.90 ± 0.60	0.003*
	**CPQ sum**	0.00/5.30 (2.90)	2.90 ± 1.33	0.40/5.80 (3.85)	3.78 ± 1.27	0.003*

^*^Significant difference as *P* ≤ 0.05

**Table 9:** Comparison between 1 st and last visit patient oral health related quality of life score within each group

		** 1 st visit**		**Last visit**		***P* value**
		**Min/Max (Median)**	**Mean ± SD**	**Min/Max (Median)**	**Mean ± SD**	
Group I (Experimental)	**Oral symptoms**	0.40/3.00 (1.80)	1.78 ± 0.75	0.00/2.40 (1.00)	1.03 ± 0.53	< 0.001*
	**Functional limitation**	0.00/3.00 (1.00)	1.07 ± 0.73	0.00/2.00 (0.60)	0.66 ± 0.53	< 0.001*
	**emotional well being**	0.00/4.00 (0.80)	1.05 ± 0.93	0.00/2.20 (0.60)	0.69 ± 0.62	< 0.001*
	**Social well being**	0.00/2.50 (0.60)	0.77 ± 0.69	0.00/1.90 (0.40)	0.52 ± 0.52	< 0.001*
	**CPQ sum**	0.80/8.70 (4.65)	4.67 ± 2.04	0.00/5.30 (2.90)	2.90 ± 1.33	< 0.001*
Group II (Control)	**Oral symptoms**	0.00/3.00 (2.00)	1.70 ± 0.89	0.00/2.60 (1.20)	1.11 ± 0.73	< 0.001*
	**Functional limitation**	0.00/3.40 (1.10)	1.35 ± 0.88	0.00/2.60 (0.80)	0.92 ± 0.72	< 0.001*
	**Emotional well being**	0.00/3.80 (1.20)	1.35 ± 0.91	0.00/2.40 (0.90)	0.84 ± 0.66	< 0.001*
	**Social well being**	0.10/2.60 (1.35)	1.28 ± 0.71	0.00/2.00 (0.90)	0.90 ± 0.60	< 0.001*
	**CPQ sum**	2.10/8.80 (5.50)	5.69 ± 1.70	0.40/5.80 (3.85)	3.78 ± 1.27	< 0.001*

^*^Significant difference as *P* ≤ 0.05

## Discussion

Molar incisor hypomineralization has received extensive global attention, with numerous studies indicating a rising prevalence of the condition. One of the most common complaints associated with MIH is hypersensitivity; therefore, this study was conducted. It specifically focused on children aged 8 to 10 years, as previous research has shown that hypersensitivity is notably higher among 8-year-olds, especially in their molars [11].

Hypersensitivity was assessed in this study using an evaporative stimulus. According to a published paper, the evaporative method is the most preferred approach for provoking pain in patients with dentin hypersensitivity, as the pain intensity is elevated with the air test among the tactile tests [12].

Pain from evaporative and thermal stimuli was estimated via the Wong-Baker FACES Pain Rating Scale. This tool is considered the easiest for children to use when expressing their pain. Additionally, high reproducibility and clinical utility have been reported for the Wong-Baker scale. The SCHIFF scale was used to measure tooth sensitivity to evaporative stimulus in this study. Previous studies indicate that the SCHIFF scale shows higher sensitivity and specificity than other assessment scales [13, 14].

The current study looked at the OHRQoL of children who had MIH. Dantas-Neta and Portella et al. found that MIH negatively affected the OHRQoL of affected children, in that they had lower OHRQoL scores than children without MIH. Furthermore, Gutiérrez et al. showed that moderate to severe MIH causes a greater negative impact on QoL than mild MIH. A systematic review regarding the effects of MIH on OHRQoL corroborates these studies and demonstrates the effect MIH has on the OHRQoL of children with MIH [15–20].

The most utilized tool for the assessment of OHRQoL in children is the Child Perception Questionnaire (CPQ); however, previous work has validated the Arabic language version of the CPQ for 8- to 10-year-old children as being both valid and reliable making it appropriate for this population. Hence, the Arabic version was used in the present study [21].

The follow-up period within this randomized clinical trial was one month; one month is the usual interval to evaluate desensitizing agents in clinical trials because it allows an adequate amount of time for the mechanisms of action of these products (e.g., occlusion of dentinal tubules, remineralization) to occur, while at the same time allowing for the assessment of early clinical results [22–24].

Statistical analysis of evaporative stimuli using the Schiff scale revealed that there are significant statistical differences in post-treatment hypersensitivity between the two groups. The first treatment delivered higher initial levels of hypersensitivity to group I participants compared to group II participants and was followed by a period of gradual decrease. Groups I and II both maintained decreased levels of hypersensitivity throughout the duration of the study.

These findings align with a randomized controlled trial in 2021 comparing the PRG barrier coat to fluoride varnish, which also indicated that the Giomer varnish resulted in an immediate reduction in hypersensitivity. This could be related to the bioactive nature of GIOMER varnish, which is attributed to the existence of PRG particles, these particles serving as filler to occlude dentinal tubules [7].

In this study on pain response to evaporative stimuli using face pain rating scales, our statistical results demonstrated a significant difference between both groups, with the experimental group (Group I) demonstrating a greater reduction in dentin hypersensitivity. Ravishankar supported these study findings in their published clinical trial in 2018, which compared the effectiveness of the Giomer varnish to two other desensitizing agents. They used an evaporative stimulus similar to this study. As their findings were similar to ours, they provide support for the Giomer varnish’s ability to provide an immediate reduction in dentinal hypersensitivity with the effect lasting one month at follow up; the time period of their study and our study were both the same [7].

Additionally, a different study also produced similar findings, indicating that the long-term benefit of Giomer varnish may be due to the resinous composition that makes it more resistant to pH loss and to mechanical removal from the occluded dentinal tubules by brushing [24].

The results showed significant statistical changes in response to pain stimuli from baseline to the end of the study among participants in both groups. The results are comparable with those of research that was done in April 2025; Giomer varnish showed the fastest effect on hypersensitivity among all other desensitizers. Moreover, it was able to maintain lower levels of hypersensitivity in the course of thermal test possibly because of the bioactive S-PRG fillers used in it. They ensure ion release that leads to dentinal tubule occlusion and potential remineralization effects. These results further validate the findings of this study [25].

Supporting the results of our study a 2026 review stated that Giomer varnish helped in Molar incisor hypo mineralization with dentin hypersensitivity due to role of bioactive potential S-PRG (surface pre-reacted glass particles)filler on dental tissues [26].

Furthermore, a controlled clinical trial published in 2023 compared the effects of Fluoride varnish to another sodium fluoride varnish in reducing dentin hypersensitivity. Fluoride varnish demonstrated a significant change in the reduction of dentin hypersensitivity in intragroup comparisons between follow-up visits, aligning with the results of our study. This indicates that fluoride varnish effectively decreases hypersensitivity and maintains its effect over the follow-up period; this could be related to multiple applications of varnish [27].

This study evaluated OHRQoL, showing that the total CPQ score was lower in the experimental group (Group 1) than in Group 2, indicating better quality of life post-treatment. This aligns with findings from a German trial on children with MIH and hypersensitivity, which showed a significant decrease in CPQ scores after treatment using the same questionnaire. These results suggest that treating hypersensitivity can improve children's OHRQoL [19].

## Limitations

The limitations of this paper include the fact that the severity of Molar Incisor Hypomineralization was not taken into consideration, as mild cases were compared to severe cases. Additionally, the follow-up period needs to be extended. The questionnaire used was challenging for some children to answer, and the application of rubber dams without anesthesia caused discomfort for most patients. Furthermore, postoperative instructions were not provided to guardians in written form; some guardians forgot the instructions.

## Recommendations

We recommend that future researchers increase the follow-up period, assess retention for both varnishes, compare fluoride release between the two varnishes, subdivide groups according to severity, simplify the questionnaire, provide written postoperative instructions for guardians, explore alternative methods for isolation that do not involve rubber dams, and to consider a multi-centered study.

## Conclusions

Based on the findings of the present study, both Giomer varnish and Fluoride varnish demonstrated effectiveness in reducing hypersensitivity associated with non-carious MIH-affected molars and improving oral health-related quality of life in children. The Giomer varnish exhibited a more favorable short-term response at selected follow-up intervals; however, these findings should be interpreted with caution considering the relatively short follow-up period and the differences in application protocols between the investigated materials. Both interventions maintained their desensitizing effects throughout the four-week follow-up period. Additionally, reductions in hypersensitivity to both thermal and evaporative stimuli were observed in both groups. Further studies with longer follow-up periods and standardized application regimens are recommended to confirm the long-term effectiveness and comparative clinical performance of these varnishes.

## Data Availability

The datasets analyzed and/or generated during this work are available from the corresponding author upon reasonable request.
